# Three-dimensional preoperative planning in the weight-bearing state: validation and clinical evaluation

**DOI:** 10.1186/s13244-021-00994-8

**Published:** 2021-04-07

**Authors:** Tabitha Roth, Fabio Carrillo, Matthias Wieczorek, Giulia Ceschi, Hooman Esfandiari, Reto Sutter, Lazaros Vlachopoulos, Wolfgang Wein, Sandro F. Fucentese, Philipp Fürnstahl

**Affiliations:** 1 Institute for Biomechanics, ETH Zurich, Leopold-Ruzicka-Weg 4, 8093 Zurich, Switzerland; 2 Research in Orthopedic Computer Science (ROCS), University Hospital Balgrist, University of Zurich, Balgrist Campus, Lengghalde 5, 8008 Zurich, Switzerland; 3 ImFusion GmbH, Agnes-Pockels-Bogen 1, 80992 Munich, Germany; 4 Department of Radiology, Balgrist University Hospital, University of Zurich, Forchstrasse 340, 8008 Zurich, Switzerland; 5 Department of Orthopedics, Balgrist University Hospital, University of Zurich, Forchstrasse 340, 8008 Zurich, Switzerland

**Keywords:** 3D preoperative planning, Weight-bearing, Osteotomy, 2D–3D registration, 3D measurement methods

## Abstract

**Objectives:**

3D preoperative planning of lower limb osteotomies has become increasingly important in light of modern surgical technologies. However, 3D models are usually reconstructed from Computed Tomography data acquired in a non-weight-bearing posture and thus neglecting the positional variations introduced by weight-bearing. We developed a registration and planning pipeline that allows for 3D preoperative planning and subsequent 3D assessment of anatomical deformities in weight-bearing conditions.

**Methods:**

An intensity-based algorithm was used to register CT scans with long-leg standing radiographs and subsequently transform patient-specific 3D models into a weight-bearing state. 3D measurement methods for the mechanical axis as well as the joint line convergence angle were developed. The pipeline was validated using a leg phantom. Furthermore, we evaluated our methods clinically by applying it to the radiological data from 59 patients.

**Results:**

The registration accuracy was evaluated in 3D and showed a maximum translational and rotational error of 1.1 mm (mediolateral direction) and 1.2° (superior-inferior axis). Clinical evaluation proved feasibility on real patient data and resulted in significant differences for 3D measurements when the effects of weight-bearing were considered. Mean differences were 2.1 ± 1.7° and 2.0 ± 1.6° for the mechanical axis and the joint line convergence angle, respectively. 37.3 and 40.7% of the patients had differences of 2° or more in the mechanical axis or joint line convergence angle between weight-bearing and non-weight-bearing states.

**Conclusions:**

Our presented approach provides a clinically feasible approach to preoperatively fuse 2D weight-bearing and 3D non-weight-bearing data in order to optimize the surgical correction.

## Key points

- Preoperative planning of orthopaedic surgeries is increasingly performed using CT-reconstructed 3D models.
- CTs are acquired in lying position and do not contain weight-bearing information.
- We suggest registering CTs with standing radiographs to obtain weight-bearing 3D models.
- Registration of 59 patients showed significant differences between non-weight-bearing and weight-bearing positions.
- 2D-3D registration is a clinically feasible method to obtain weight-bearing 3D models.

## Background

Malalignment of the lower limb often causes a shift of the load-bearing axis away from the center of the knee, leading to an imbalance in load distribution within the knee joint. Various studies have demonstrated correlations between the aforementioned malalignment and the development of knee osteoarthritis (OA) [1, 2], including not only chondral damage but also alterations in bony microstructure of the underlying subchondral trabecular bone [3, 4]. While progressed degeneration requires surgical treatment through unicompartmental or total knee replacement (TKR) [5], a joint-preserving realignment surgery achieved through a corrective osteotomy is the benchmark in younger patients with unilateral OA [6]. In a corrective osteotomy intervention, the pathologically deformed bones are cut, realigned and subsequently fixed with an orthopedic implant, transferring the load from the pathological to the healthy compartment of the knee. Precise postoperative alignment to a neutral weight-bearing axis or even mild over-corrections have been found to result in improved functional scores [7]. To achieve this, accurate preoperative planning and radiographic measurements are crucial.

Traditionally, realignment surgeries were planned in two dimensions (2D) using long-leg standing radiographs in order to realign the loaded region and achieve an optimal joint alignment, which is relevant to counteract OA development [8, 9]. Technical advances of the recent years gave rise to modern computer-assisted surgery methods that are based on 3D models of the patient anatomy [10, 11]. These 3D models are generated from CT scans, which are acquired in a supine, non-weight-bearing position. By using these models, potential inaccuracies can occur in the preoperative plan that are attributed to the non-weight bearing position of the subject during the image acquisition, but one would need to make this compromise to gain the ability of planning in 3D. We have recently shown that the relevant radiological metrics in the context of preoperative planning, such as the mechanical axis (MA) and the joint line convergence angle (JLCA), are significantly different between 2D weight-bearing and 2D non-weight-bearing as well as between 2D non-weight-bearing and 3D non-weight-bearing conditions [12].

In the current clinical practice, there are no validated computer methods that allow for 3D preoperative planning of corrective osteotomies under weight-bearing conditions. We suggest to address this gap by implementing an algorithm to register the 3D non-weight-bearing imaging data to the 2D weight-bearing counterparts. In orthopedics, 2D-3D registration has already been widely used in various applications, such as in kinematic analyses, image-guided interventions or postoperative estimation of implant position [13–20]. For example, algorithms have been developed to accurately estimate the 3D pose of prosthesis components after TKR from single fluoroscopy images. These algorithms, which are from the feature-based registration family, solve the registration problem by iteratively projecting the contours of the 3D surface model onto the 2D image and minimizing the discrepancies between the projected contours and the actual 2D contours [14, 15].

Similarly, 2D-3D registration algorithms relying on single-plane fluoroscopy images were applied to study tibiofemoral kinematics during static and dynamic weight-bearing postures. While some groups also used feature-based approaches [16, 17], more recent publications describe so-called intensity-based algorithms [18–20]. In such techniques, synthetic 2D images called Digitally Reconstructed Radiograph (DRRs) are first obtained from the volumetric CT scans placed in an initial position. Later, the CT scan is iteratively transformed into a new position from which new DRRs are generated. A similarity metric is calculated at each point between the DRR and the actual 2D radiograph and the global optima transformation is found at a point resulting in the highest similarity. However, the out-of-plane registration error in all of these studies were shown to be too large for the purpose of 3D preoperative planning. Additionally, these approaches only focus on the knee joint itself and do not consider the alignment of the entire leg.

For the purpose of preoperative planning of corrective osteotomies, the translation and rotation in the coronal plane is of particular interest. Using biplanar long-leg standing radiographs as a registration target is therefore a reasonable choice to maximize registration accuracy also in the frontal plane. Fuji et al. [21] used one X-ray source in combination with a rotational table, which was positioned at 0° and 60° relative to the optical axis of the X-ray source. Calibration for the two positions was performed manually beforehand.

Specialized centers are increasingly replacing conventional radiographs with the EOS imaging system (EOS imaging system, EOS, Paris, France), a pre-calibrated imaging modality capable of providing biplanar low-dose standing long-leg radiographs. This system utilizes two synchronized x-ray beams, perpendicular to one another, which scan the subject from top to bottom in a vertical movement. The EOS device therefore has a different imaging geometry than conventional projective radiographs [22].

Given the clinical evidence that malalignment along with its associated change in load distribution are independent risk factors for OA progression [23], the goal of our study was to develop a pipeline that allows for 3D preoperative planning under weight-bearing conditions. To this end, we leveraged a 2D–3D registration algorithm with biplanar EOS 2D images as the registration target to transform the 3D models from a non-weight-bearing to a weight-bearing posture. Thereafter, we validated the pipeline in terms of registration accuracy and evaluated its clinical feasibility in a retrospective study.

## Methods

**Fig. 1:**
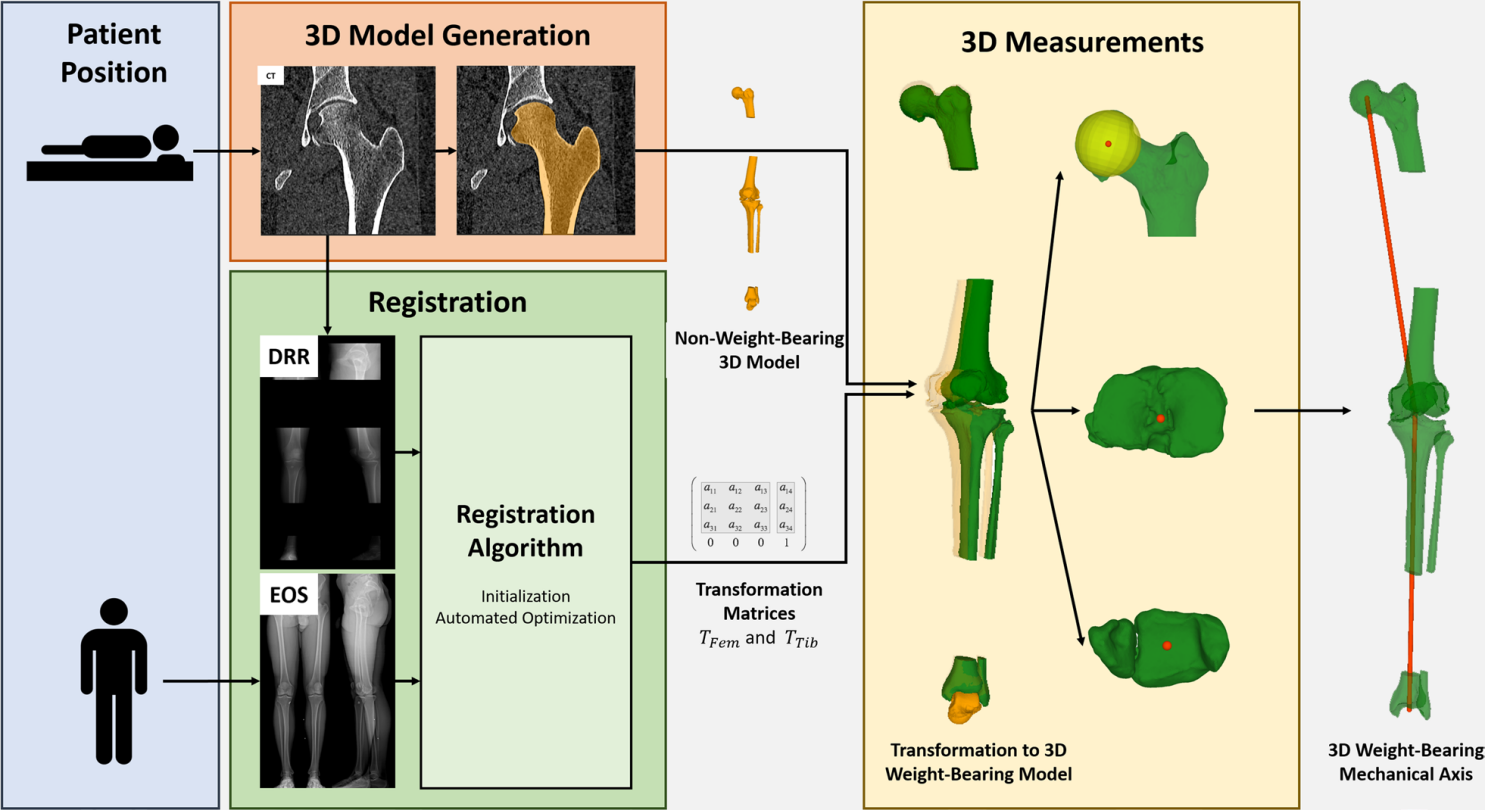
Registration and measurement pipeline for the 3D preoperative planning of corrective osteotomies under weight-bearing conditions. CT scans of the hip, knee and ankle joints as well as biplanar EOS images are required as an input for the pipeline. MA and JLCA assessed in a weight-bearing position are the output

The method section is organized in three parts. In the first part, we describe our developed registration and measurement pipelines capable of transferring 3D models into a weight-bearing posture and assess the MA as well as the JLCA in 3D (Fig. 1). As the input to our model, we use CT scans of the hip, knee and ankle joints as well as biplanar EOS images of the entire leg. As the output, the user receives 3D MA and JLCA measurements assessed in a weight-bearing posture.

In the second part, the accuracy of the registration algorithm is calculated in the form of a quantitative validation. Lastly, the clinical feasibility of our approach was evaluated retrospectively by applying our pipeline to a larger radiological dataset of patients who underwent corrective osteotomy of the leg.

### 3D model generation module

Segmentation of the CT data was performed using a commercial segmentation software (Mimics Medical 19.0, Materialise NV, Leuven, Belgium). 3D models were generated using the Marching Cube algorithm [24] and were represented in the form of triangular surface meshes (stereolithographic models; STL) as described by [25]. The femoral and tibial segments were then transformed to a weight-bearing state by applying the transformation matrices obtained from the registration module (described below). The transformation of the 3D segments was done in Matlab (Matlab 2019a, The MathWorks Inc., Natick, MA, USA).

### Registration module

**Fig. 2:**
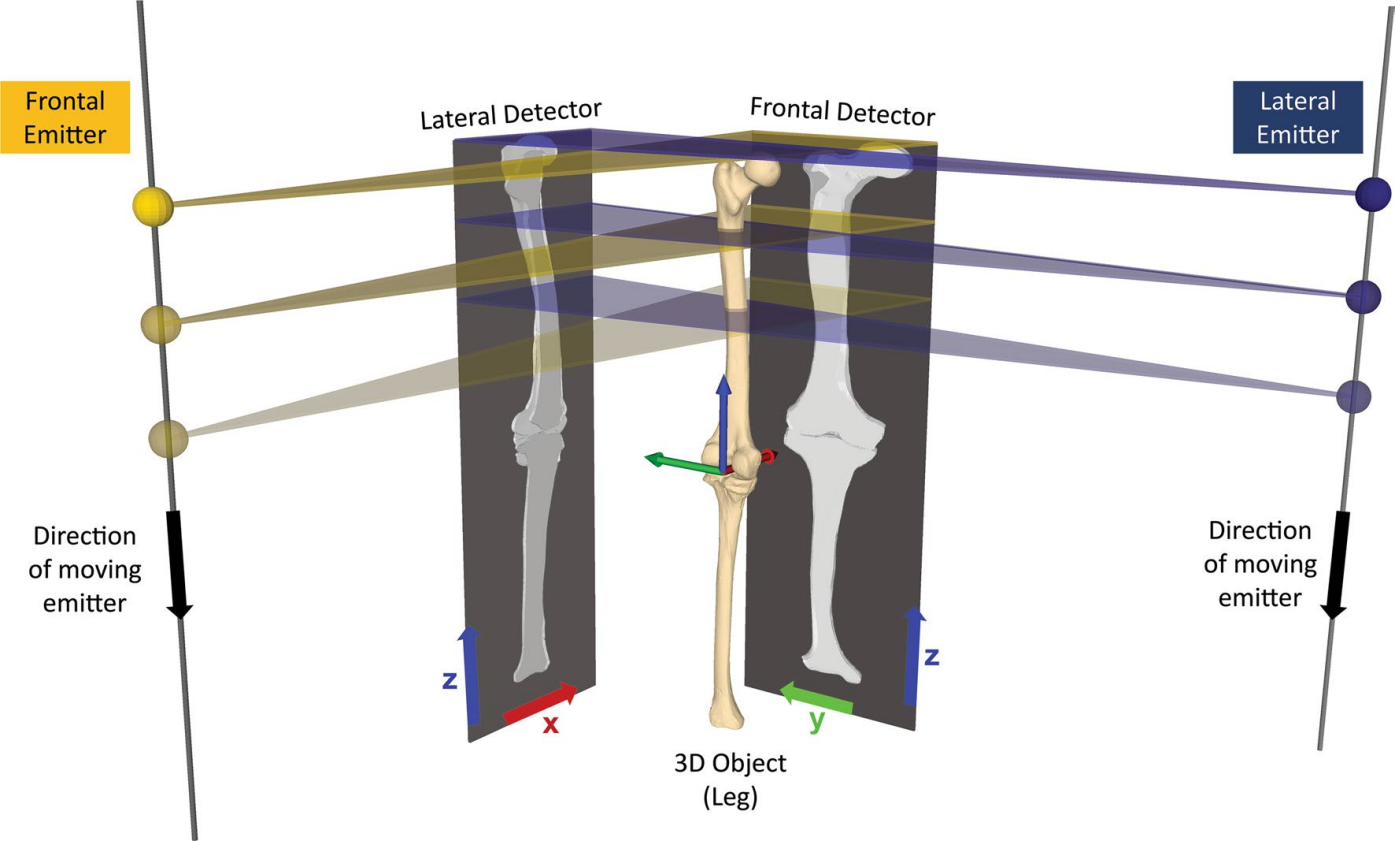
Geometry of the EOS System. The EOS imaging systems produces biplanar long-leg standing X-rays by using a fan-beam scan approach, in which only the horizontal axis of the image is affected by projective effects. Frontal (in yellow) and lateral (in blue) emitters are depicted as spherical sources. The emitters move synchronously along the z-axis (shown in green). The vertical y-axes of the lateral and frontal detectors directly relate, without magnification, to the z-axis in the EOS coordinate system

The registration algorithm finds the optimal position by comparing intensity values between the EOS radiographs and DRRs [27], generated from CT and using the same geometry as the EOS system. Contrary to a standard C-arm x-ray system, the EOS system uses a fan-beam scan approach in which only the horizontal axes of the images (x and y) are affected by projective effects, introducing magnification in this direction (Fig. 2). The vertical z-axis of the image on the other hand directly relates, without magnification, to the z-axis in the world coordinate system. In order to mitigate the magnification effect on the horizontal axes, the EOS system rescales the images such that the they are virtually moved to a detector which is placed at the isocenter of the system, i.e. within the patient. All information required to reconstruct the EOS geometry were retrieved from the DICOM tags of the EOS images. A detailed description of the geometry can also be found in [22].

**Fig. 3:**
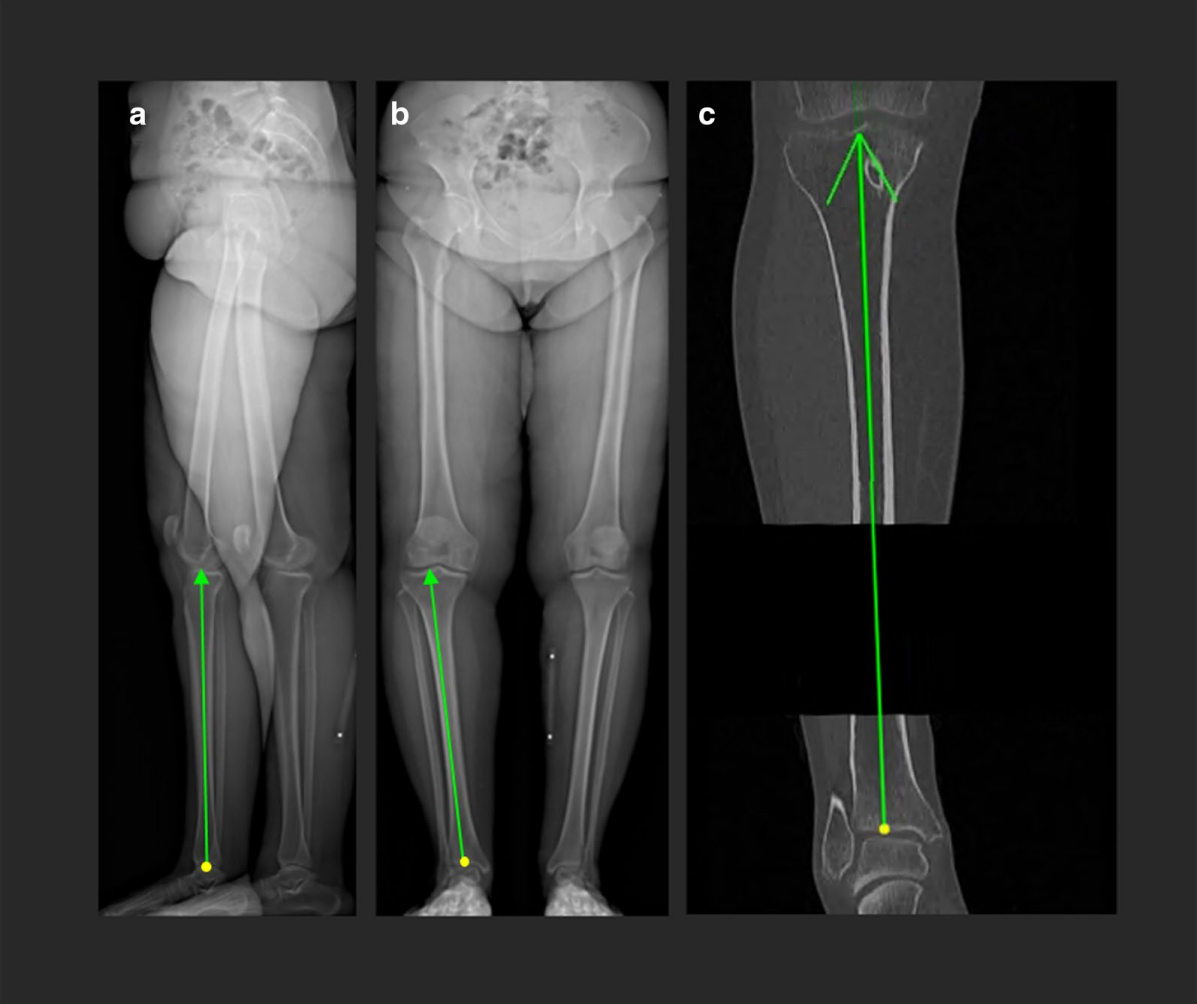
Manual annotations to initialize the registration algorithm. Three user selected annotations are used to find the initial position between EOS and DRR. In this example, an arrow is drawn from the distal to the proximal end of the tibia in the two EOS images (**a**, **b**) as well as the CT scan (**c**)

The EOS images as well as the CT served as the input for the registration algorithm, which was used to individually register femur and tibia to the EOS images. Two pre-processing steps were required for the algorithm: First, the registration was initialized based on three user-selected annotations. An arrow connecting the same two anatomical landmarks was drawn in both the frontal and lateral EOS radiographs (2D) as well as the CT scan (3D) (Fig. 3). For example, the three arrows for the tibia were drawn from the distal end of the tibia to the center of the tibial plateau in all three images. Based on the aforementioned EOS geometry, the 2D image coordinates of the start and end points of the EOS arrow annotations (frontal and lateral) were subsequently triangulated, i.e. the 3D coordinates of the start and end point were reconstructed from their frontal and lateral 2D projections. The resulting 3D coordinates in the EOS space were used together with the 3D coordinates of the arrow annotation in the CT space (also connecting the condyle midpoint with the center of the femoral head) to apply an initial transformation on the entire CT volume so that its projection on the EOS planes (DRRs) were roughly congruent with the EOS images (i.e. to initialize the 2D-3D registration process).

In the second pre-processing phase, the user had to draw a rectangular mask around the respective bone (femur or tibia) in both planes of the EOS in order to define the desired region of interest in which the registration was conducted.

Following the notion of intensity-based 2D-3D registration, our registration was achieved through an optimization routine, through which the pose of the CT scan was optimized with respect to a six degrees-of-freedom transformation (3 Euler angles and 3 translational parameters), resulting in a 4 × 4 transformation matrix *T*. After each optimization step, new DRRs were generated using the updated CT pose and subsequently compared with the corresponding EOS images. As the cost function, we use the sum of a variance-weighted localized normalized cross correlation (LNCC) similarity with a patch size of nine pixels between the simulated DRR and the corresponding EOS images [28]. The optimization executed using a bound optimization by quadratic approximation (BOBYQA) algorithm [29] from the nlopt library that is relatively fast and does not require the calculation of cost function derivatives [30].

The output of the registration algorithm is in the form of a 4 × 4 transformation matrix, combining a 3 × 3 rotation matrix and a 1 × 3 translation vector, describing the relative transformation from the 3D coordinate system of the EOS to the coordinate system of the CT.

Registration is performed individually for the femoral and the tibial segments, resulting in the transformation matrices $T_{{{\text{EOS}}}}^{{{\text{CT}}}} ({\text{FEM}})$ and $T_{{{\text{EOS}}}}^{{{\text{CT}}}} ({\text{TIB}})$, respectively.

The registration algorithm was implemented within the ImFusion Suite software environment (ImFusion GmbH, Munich, Germany).

### 3D measurement module

Once the registration is completed, the non-weight-bearing 3D models of femur and tibia were transformed using $T_{{{\text{EOS}}}}^{{{\text{CT}}}} ({\text{FEM}})$ and $T_{{{\text{EOS}}}}^{{{\text{CT}}}} ({\text{TIB}})$, respectively, and used as a representative of the weight-bearing posture. To this end, a 3D measurements module was developed that took the registered 3D models as input in order to calculate MA and JLCA under weight-bearing conditions.

**Fig. 4:**
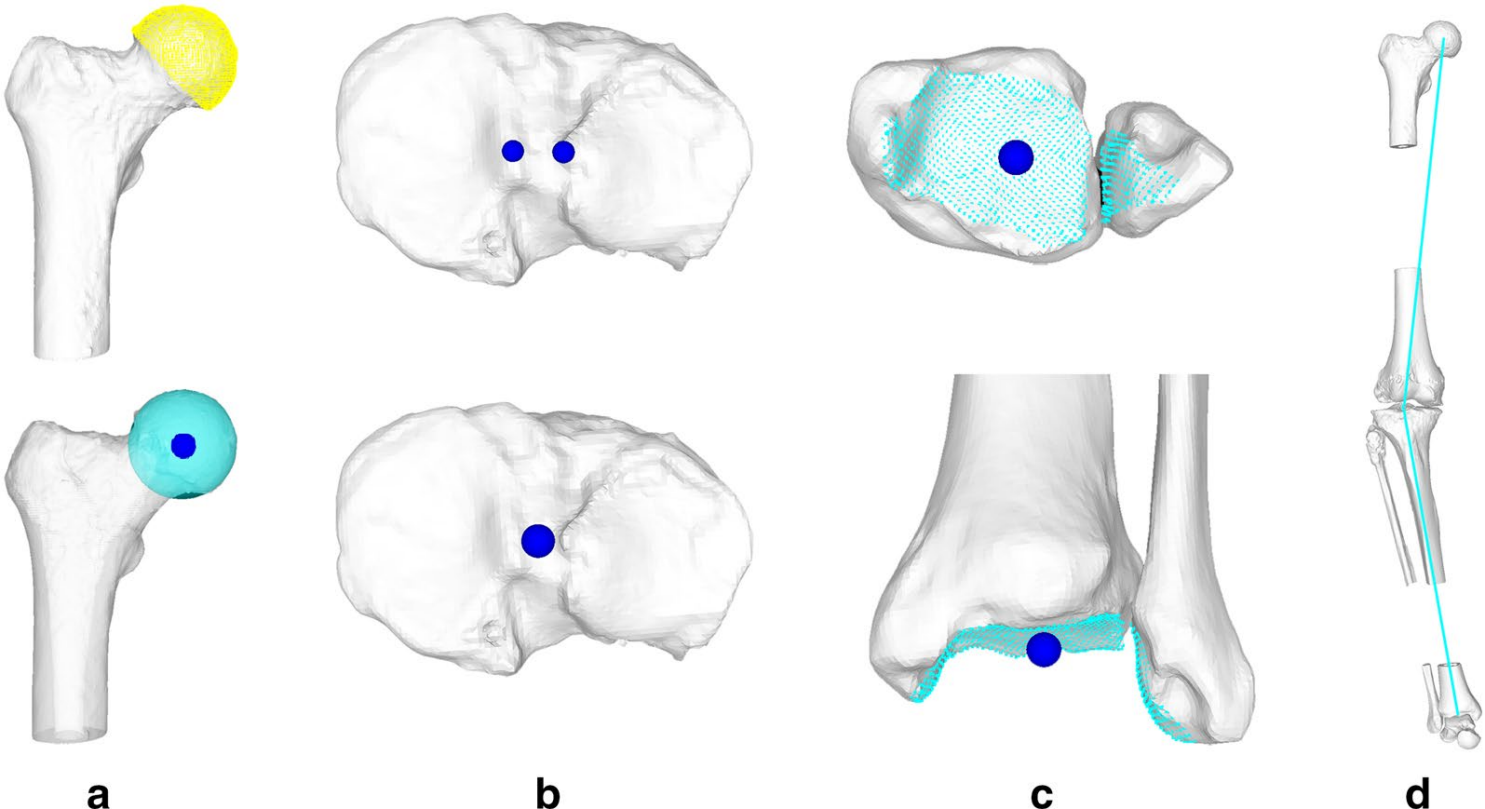
Calculation of the mechanical axis in 3D. **a** The HJC is defined as the center of a sphere fitted to the femoral head. **b** The KJC is located in between the intercondylar eminences on the tibial plateau. **c** The AJC is defined as the center of all points of the distal tibial and fibular articular surfaces

First, the hip, knee and ankle joint centers were determined as described in [11]. The hip joint center (HJC) was defined as the center of a sphere, fitted to the user-selected points of the femoral head using least-square regression [31] (Fig. 4a). The knee joint center (KJC) was defined as the midpoint between the intercondylar eminences of the tibial plateau (Fig. 4b). Thereafter, the ankle joint center (AJC) was determined as the center of all points belonging to the distal articular surfaces of the femoral and the tibial segments. These points on the articular surface were found by calculating the closest-point distance to the talus model and considering only points below a user-defined distance threshold. The threshold is visually determined for each case until the entire articular surface is selected (Fig. 4c).

Using these landmarks, the 3D leg models were reoriented in a right-handed anatomical coordinate system. The mechanical leg axis (HJC to AJC) was aligned perpendicular to the axial plane (superior direction along the positive y-axis), and subsequently rotated along this axis until the anterior surface of the patella was parallel to the frontal plane (anterior direction along the positive z-axis). The MA and JLCA were assessed in 3D using the methods as follows. Varus MA and laterally opened JLCA were denoted as positive, valgus MA and medially opened JLCA as negative.

The MA was assessed as described in [11] based on the hip joint, knee joint and ankle joint centers (Fig. 4d). Given the centers, the MA was calculated as the angle between a line connecting the HJC and the KJC and a second line between the KJC and AJC, projected to the frontal plane.

**Fig. 5:**
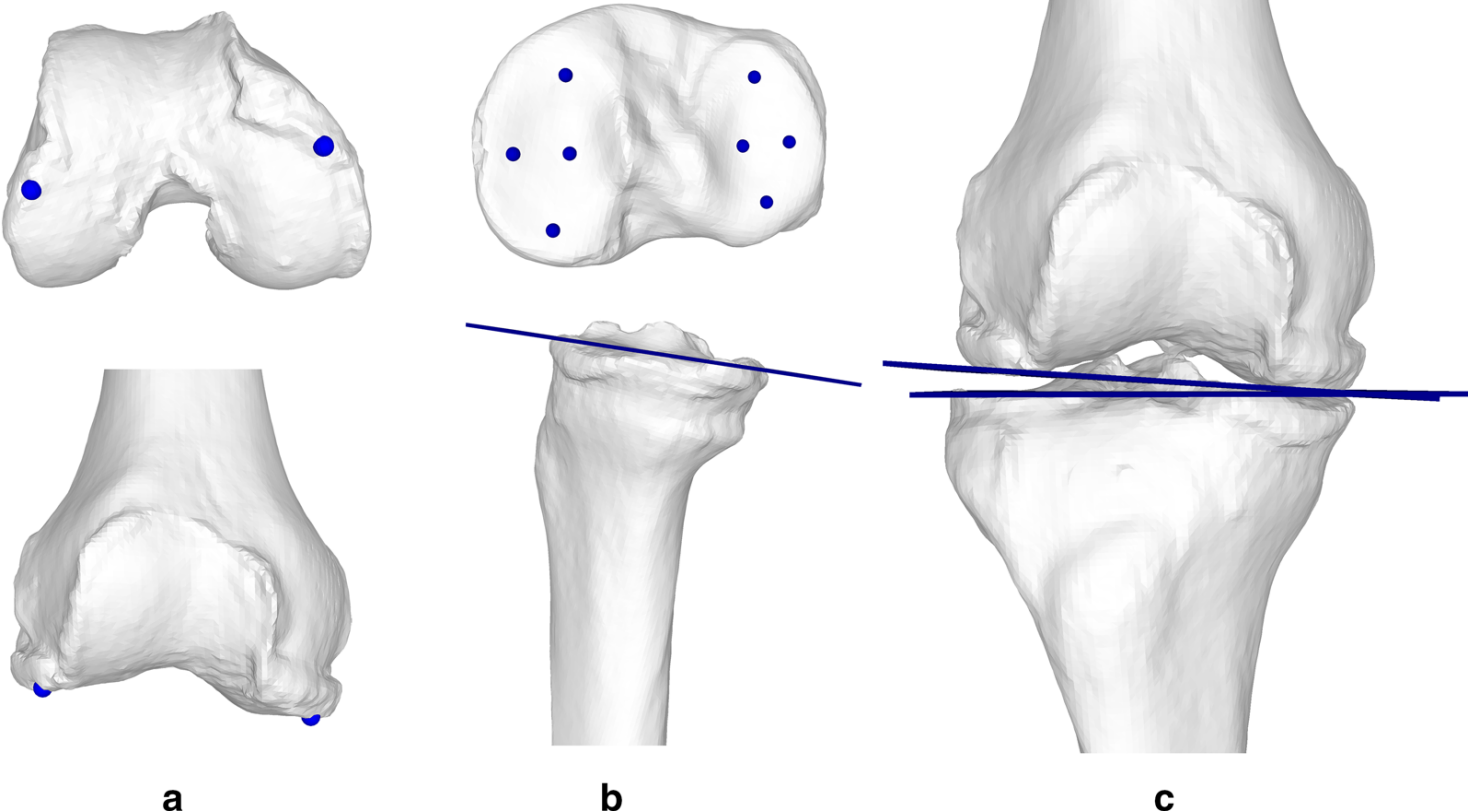
Calculation of the joint line convergence angle in 3D. **a** The FCT is defined by the two most distal points, one on each condyle. **b** The TCT is found by defining the tibial plateau plane using user selected points on the plateau. C Both the FCT and the tibial plateau plane are projected to the frontal plane to find the JLCA

To measure the JLCA, the longitudinal axis of the femur was determined by applying principal component analysis on all points of the bone model [31]. The distal femoral epiphysis was then isolated by cutting the femur right above the femoral epicondyles. A k-means algorithm [32], initialized as in [33], was used to automatically divide the epiphysis into the two condyles. The three most distal points along the longitudinal axis were found for each condyle cluster (Fig. 5a), and the femoral condyle tangent (FCT) was drawn by connecting their means.

The tibial condyle tangent (TCT) was defined as the frontal projection of the tibial plateau plane. The tibial plateau plane was defined using a least squares approach [31], minimizing the distance to eight user-selected points, arranged as rhomboids on the tibial plateaus (Fig. 5b). Finally, the JLCA was defined as the angle between the frontal projection of the FCT and the TCT (Fig. 5c). These measurements were performed using our in-house planning software CASPA (CASPA 5.32, University Hospital Balgrist, Zurich, Switzerland) and Matlab (Matlab 2019a, The MathWorks Inc., Natick, MA, USA).

### Validation

The accuracy of the registration module was assessed using a leg phantom (full leg 1132–48, Sawbones, Vashon Island, USA). The phantom was rigidly mounted on a wooden frame and each bone was equipped with a set of six radiopaque spherical markers of 3 mm diameter, visible both in the CT volume and the EOS radiographs. The validation medium was scanned using a CT scanner with a slice thickness of 0.6 mm and an in-plane pixel spacing of 0.26 mm (Somatom Definition AS Siemens Healthcare, Erlangen, Germany). Subsequently, two orthogonal long-leg standing radiographs were acquired using the EOS system.

To obtain the ground truth transformation for the validation setup, we first calculated the 3D coordinates of each marker in the CT scan’s coordinate system ${\text{CS}}_{{{\text{CT}}}}$ by manually segmenting the CT scan to acquire a 3D model of the marker. The centers of the resulting 3D markers were found using a sphere-fitting algorithm [34].

Later, the 3D coordinates $\left( {P_{x} ,P_{y} ,P_{z} } \right)$ of the center of each marker were determined with respect to the 3D EOS coordinate frame $CS_{{{\text{EOS}}}}$ based on their projections in the frontal and lateral EOS images using the following pair of linear equations:

$$\left( {\begin{array}{*{20}c} { - h_{f} } & {f_{f} } \\ {f_{l} } & { - h_{l} } \\ \end{array} } \right)\left( {\begin{array}{*{20}c} {P_{x} } \\ {P_{y} } \\ \end{array} } \right) = \left( {\begin{array}{*{20}c} {f_{f} } & {h_{f} } \\ {f_{l} } & {h_{l} } \\ \end{array} } \right)$$

$$P_{z} = z_{0} - \lambda_{z} \left( {\frac{{v_{l} + v_{f} }}{2}} \right)$$

where *h*_*f*_ and *h*_*l*_ correspond to the horizontal position of the landmark in image (pixel) space in the frontal and lateral planes, respectively. *f*_*f*_ = 918 mm and *f*_*l*_ = 918 mm correspond to the distance of the frontal and lateral emitter to the EOS isocenter. Additionally, $z_{0} = 477.5\,{\text{mm}}$ is the initial height of the emitter, $\lambda_{z} = 0.179363\,{\text{mm}}$ is the pixel pitch of the image in the vertical direction and *v*_*l*_, *v*_*f*_ are the vertical position of the landmark in image (pixel) space in the lateral and frontal planes. More details regarding the EOS reconstruction geometry can be found in [22].

Once the 3D EOS coordinates of each marker were determined for each bone, we applied an iterative closest point (ICP) registration algorithm [35] to register the set of twelve corresponding 3D marker coordinates, to find the ground truth transformation matrix between the 3D CT and the 3D EOS coordinate sets (${\text{GT}}_{{{\text{EOS}}}}^{{{\text{CT}}}}$).

CT and EOS radiographs were then registered using the previously described registration module, individually for tibia and femur, resulting in the transformation matrices $T_{{{\text{EOS}}}}^{{{\text{CT}}}} ({\text{Fem}}),T_{{{\text{EOS}}}}^{{{\text{CT}}}} ({\text{Tib}})$.

Finally, in order to validate our registration method, the registration error was obtained as $E_{{{\text{Fem}}}} = T_{{{\text{EOS}}}}^{{{\text{CT}}}} ({\text{Fem}})^{ - 1} *{\text{GT}}_{{{\text{EOS}}}}^{{{\text{CT}}}}$ for the femur, and $E_{{{\text{Tib}}}} = T_{{{\text{EOS}}}}^{{{\text{CT}}}} ({\text{Tib}})^{ - 1} *GT_{{{\text{EOS}}}}^{{{\text{CT}}}}$ for the tibia.

Our right-handed coordinate systems were oriented with the x-axis to the left, while the y- and z-axes correspond to the posterior and superior directions, respectively.

### Clinical feasibility study on patient data

For the clinical feasibility assessment, we included patients treated in our institution who underwent a corrective osteotomy procedure at either distal femur and/or proximal tibia between January 2015 and May 2020. Patients with an incomplete radiological dataset were excluded, resulting in a total of 55 patients, of whom 4 were operated on both legs. The mean age of the patients at the time of surgery was 43.5 ± 8.4 years, with 29 left and 30 right leg operation, and a gender distribution of 15 females and 44 males.

A full radiological dataset consisted of the following:

- A biplanar standing long-leg radiograph (EOS imaging system, EOS, Paris, France)
- A CT scan (Philips Brilliance 64, Philips Healthcare, Best, The Netherlands, or Somatom Definition AS Siemens Healthcare, Erlangen, Germany) according to the MyOsteotomy CT protocol (Medacta SA, Switzerland). Thereby, the hip, knee and ankle joints were scanned while the midshaft regions were skipped in order to reduce radiation exposure. Slice thickness was 1 mm with a spacing of either 0.5 or 0.8 mm, while in-plane resolution was either 0.2 or 0.4 mm.

The 3D preoperative planning was then performed for all the 59 legs using our registration and measurement pipeline, resulting in transformation matrices for femur and tibia, respectively.

All registrations were performed by the same reader. Out of the 59 cases, 20 were randomly selected and registered by a second reader in order to assess inter-rater variability.

Asymptotic Wilcoxon tests were applied (IBM SPSS for Windows, version 26, Armonk, NY, USA) to analyze the differences of means between weight-bearing and non-weight-bearing values. Mean absolute differences (MAD) were calculated between weight-bearing and non-weight-bearing states. The correlation between MADs of JLCA and MA were assessed using the Pearson correlation coefficient. The interrater reliability was calculated with the intra-class correlation coefficient (ICC, two-way random model of single measures). Tests were evaluated using a significance level of *p* ≤ 0.05.

## Results

### Validation

The translation error between ground truth transformation (${\text{GT}}_{{{\text{EOS}}}}^{{{\text{CT}}}}$) and implemented registration ($T_{{{\text{EOS}}}}^{{{\text{CT}}}} ({\text{Tib}})$ and $T_{{{\text{EOS}}}}^{{{\text{CT}}}} ({\text{Fem}})$) was 1.1, 0.3 and -0.1 mm and 0.0, 0.5 and -0.4 mm for femur and tibia in x, y and z direction, respectively. This results in Euclidean distances of 1.1 mm and 0.6 mm for femur and tibia. The rotation errors were estimated to be 0.0°, 0.1° and 1.2° for the femur and 0.0°, -0.2° and 1.1° for the tibia around x-, y- and z-axes.

### Evaluation of clinical feasibility

The registration and measurement pipeline could be successfully applied for all patients.

Mean absolute difference between weight-bearing and non-weight-bearing in 3D was 2.1 ± 1.7° (range 0°–6.2°) for the MA. MA was statistically significantly different between weight-bearing and non-weight-bearing states (means 5.4 vs. 6.1° for non-weight-bearing vs. weight-bearing, z = -2.26, *p* = 0.02). 22 out of 59 patients (37.3%) had a difference of 2° or more.

**Fig. 6:**
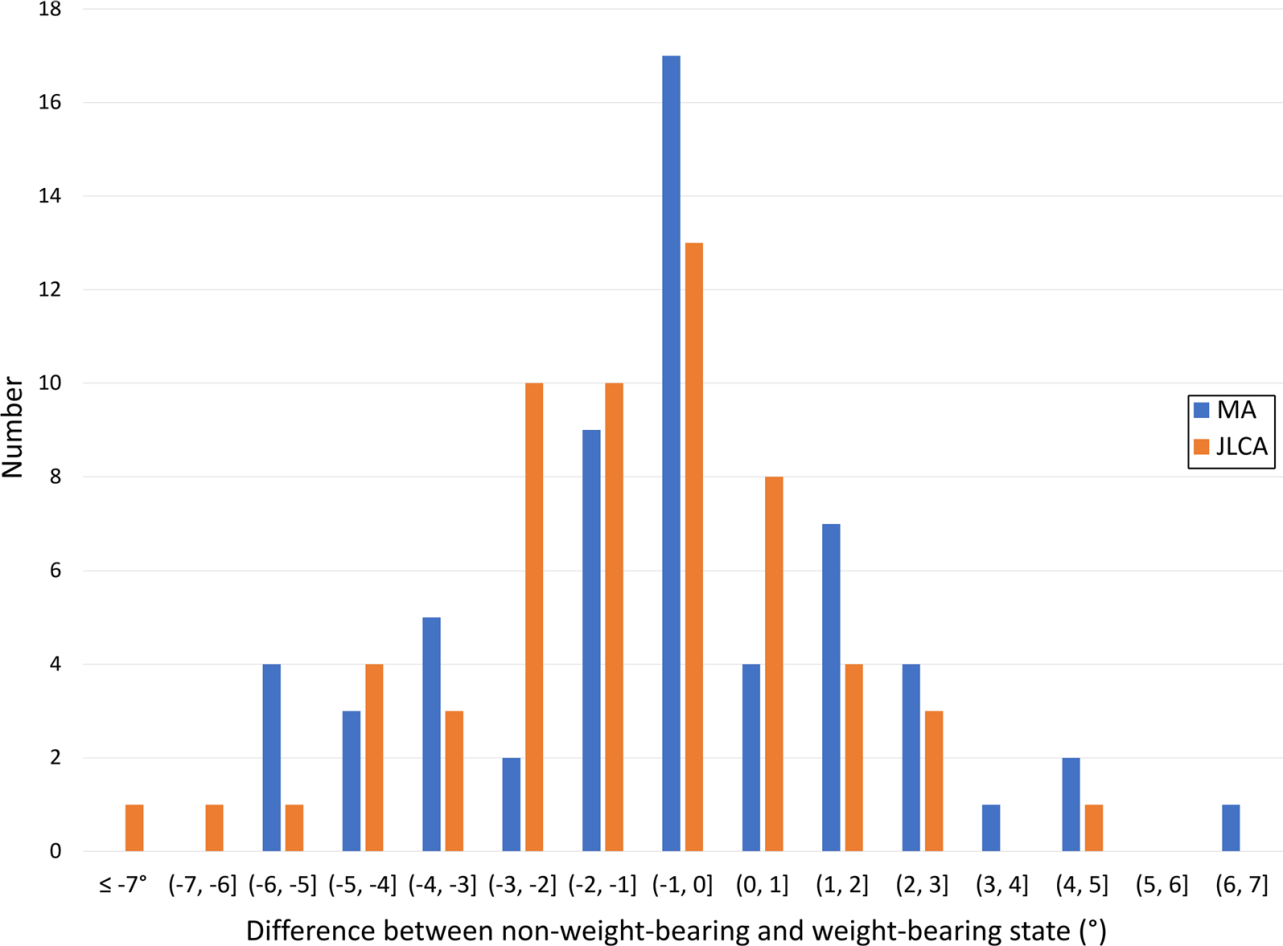
Distribution of differences between non-weight-bearing and weight-bearing states for MA and JLCA. 37.3% and 40.7% of all patients have differences of 2° or more between weight-bearing and non-weight-bearing positions for MA and JLCA, respectively

Mean absolute difference between weight-bearing and non-weight-bearing JLCA was 2.0 ± 1.6° (range 0.1°–7.5°). JLCA was statistically significantly different between weight-bearing and non-weight-bearing (means 3.6 vs. 4.9° for non-weight-bearing vs. weight-bearing, z = -3.89, *p* < 0.001). 24 out of 59 patients (40.7%) had a difference of 2° or more (Fig. 6).

The correlation coefficient for MADs of JLCA and MA was 0.752 (*p* < 0.001).

The ICC for interrater reliability was 0.961 (95% CI 0.902–0.984, *p* < 0.001).

## Discussion

This study aimed at the validation and clinical evaluation of a registration and measurement pipeline for enabling the 3D preoperative planning of corrective osteotomies in a weight-bearing state. 3D preoperative surgery planning has become increasingly important, and we have therefore recently developed and validated 3D measurement methods to assess lower limb anatomical deformities [12]. In line with our results, it has been shown that deformities are significantly underestimated when assessed on CT-based 3D models [36], which will likely lead to under-correction during surgery. In this case, the load is not transferred to the contralateral compartment but remains on the osteoarthritic side. Studies have demonstrated that under-correction is significantly correlated with high failure rates [37] and poor clinical outcome when compared to accurate correction or a slight overcorrection [38]. On the other hand, adequate axis correction creates an environment that allows for partial or complete cartilage regeneration on the femoral condyles and tibial plateau [39, 40].

Our registration algorithm was successfully applied for all patients including cases with heavily deformed bones. The entire registration pipeline for one patient took approximately 10–15 min, whereby the automated optimization only represents a few seconds. The remaining time is used to load the images and perform the manual initialization as well as the visual control of the registration result. In our experience, the automatic pose optimization is reliable and rarely requires correction by the user. If required, the user has the possibility to adjust the translational and rotational parameters of the current position and subsequently restart the automated optimization.

Our validation has shown a maximal error of 1.1 mm in translation (medial–lateral direction) and in 1.2° rotation around the superior-inferior axis. Given the fact that the registration target only consists of frontal and lateral X-rays, the slightly higher rotational errors around the vertical axis are understandable. Otherwise, the rotational errors of 0.0°–0.2° are comparable to what was reported in a related study (0.1°–0.3°) [41]. Similarly, our translational errors were close to the results reported in the same study (0.1–0.5 mm) with the exception of the 1.1 mm we have found for the femur in x-direction.

These errors are within a range that we consider to be clinically acceptable. However, the time of up to 15 min per patient might hinder the implementation of this pipeline into the everyday clinical workflow. In our future work, we aim to implement an automated initialization by using deep learning networks for automated landmark detection. Given that studies have shown a detection accuracy of less than 1 mm, it is reasonable to assume that this approach will help to find an initial overlapping between EOS and DRRs that is within the capture range of the algorithm. Furthermore, automated landmark detection will also enable the automated assessment of the anatomical deformity in 3D and therefore obviate the need for manual 3D measurements.

Our study is limited by several factors. First, our validation was only based on one sample. Furthermore, compared to the validation performed under laboratory conditions, additional challenges can be expected in a clinical setting that might influence the outcome of the registration. The registration accuracy in a clinical setting is strongly dependent on and limited by the quality of the EOS scans. Ideally, one leg is positioned slightly in front of the other in order to make both of them completely distinguishable in the sagittal scan. Notably more manual adjustment was required during the registration process in the cases with parallel leg position in the X-ray images. However, the patient should also equally distribute the weight between both legs, and knee flexion and the internal or external rotations should be kept minimal. This position can be challenging for patients with knee pain and can differ slightly from normal double-leg standing. Besides, the registration process is still partly dependent on the user and therefore not completely automated. However, since interrater reliability was very high, we assume that this does not affect the precision significantly.

## Conclusions

A 2D-3D registration pipeline was developed and validated in order to transform non-weight-bearing 3D models of the lower limb into a weight-bearing state and therefore enables 3D preoperative planning under loaded conditions. Significant differences between weight-bearing and non-weight-bearing states in the clinical application have shown that it is important to consider the effects of weight-bearing in 3D preoperative planning. The presented registration and measuring pipeline is easy to use and could be implemented into the clinical workflow.

## Data Availability

The dataset analyzed during the current study are not publicly available due to individual privacy reasons but are available from the corresponding author upon reasonable request.

## Abbreviations

AJC: Ankle joint center
BOBYQA: Bound optimization by quadratic approximation
CT: Computer tomography
DRR: Digitally reconstructed radiograph
FCT: Femoral condyle tangent
HJC: Hip joint center
ICC: Intraclass correlation coefficient
ICP: Iterative closest point
JLCA: Joint line convergence angle
KJC: Knee joint center
LNCC: Localized normalized cross correlation
MA: Mechanical axis
MAD: Mean absolute difference
OA: Osteoarthritis
STL: Stereolithographic
TCT: Tibial condyle tangent
TKR: Total knee replacement
